# Supplementary material for: The role of online peer support in pregnancy: A scoping review
Source: PLoS One. 2026 Jan 2;21(1):e0339876. doi: 10.1371/journal.pone.0339876 (PMC12758765; doi:10.1371/journal.pone.0339876)
Supplement: S2 File — (DOCX) [file pone.0339876.s002.docx]

| CONCEPT 1 | **AND** | CONCEPT 2 | **AND** | CONCEPT 3 |
| --- | --- | --- | --- | --- |
| Online Social Network |  | Peer Support |  | Pregnancy |
| **OR** |  | **OR** |  | **OR** |
| Online Community |  | Peer Group |  | Gestation |
| **OR** |  | **OR** |  | **OR** |
| Online Platform |  | Social Group |  | Prenatal |
| **OR** |  | **OR** |  | **OR** |
| Internet-based |  | Social Support |  | Antenatal |
| intervention |  | **OR** |  | **OR** |
| **OR** |  | Psychosocial support |  | Perinatal |
| Web-based |  | **OR** |  | **OR** |
| **OR** |  | Community support |  | Expectant mother |
| Social media |  | **OR** |  | **OR** |
| **OR** |  | Support group |  | Postnatal |
| Social networking site |  | **OR** |  | **OR** |
| **OR** |  | Group counselling |  | Postpartum |
| Facebook |  | **OR** |  | **OR** |
| **OR** |  | Peer counselling |  | Childbearing |
| WhatsApp |  | **OR** |  | **OR** |
| **OR** |  | Buddy |  | Childbirth |
| Twitter  **OR**  Forum  OR  Chat |  |  |  |  |

S1- Table 1 Search strategy
