# Supplementary material for: The role of online peer support in pregnancy: A scoping review
Source: PLoS One. 2026 Jan 2;21(1):e0339876. doi: 10.1371/journal.pone.0339876 (PMC12758765; doi:10.1371/journal.pone.0339876)
Supplement: S3 File — (DOCX) [file pone.0339876.s003.docx]

## Supplementary file 3

## COVIDENCE DATA EXTRACTION TOOL General Information

**Authors**
Surnames to be used.
More than two authors will be '[Surname of 1st Author] et al'

|  |
| --- |

**Title of Paper**

|  |
| --- |

**Publication**
Year of publication and Journal published

|  |
| --- |

**Year Data collected**
or the dates the data was collected if available

|  |
| --- |

## Source Origin / Country of origin

**Country** data was collected. i.e. country, city, region etc

|  |
| --- |

## Study Characteristics

**Topic**

| ☐ | Breastfeeding |
| --- | --- |
| ☐ | Weight Management |
| ☐ | Well-being |
| ☐ | Disease Support |
| ☐ | Substance Abuse and/or Drug Use |
| ☐ | General Health and/or Advice |
| ☐ | Other |
|  | |

**Aims/Purpose of the study**

|  |
| --- |

**Study Design**
i.e. Randomised-controlled trial, quasi-experimental, retrospective, longitudinal, before-after, cross-sectional etc

|  |
| --- |

**Methodology**

| ☐ | Quantitative |
| --- | --- |
| ☐ | Qualitative |
| ☐ | Mixed-Methods |
| ☐ | Other |

Is the study Interventional or Observational?

| ☐ | Interventional |
| --- | --- |
| ☐ | Observational |

**Intervention Type**
Definitions:
Discussion forum includes discussion boards.
Web/smartphone applications include interactive groups within a phone app or web application.
Social media platform group includes groups on social media platforms such as Facebook, WhatsApp etc.
SMS Text messaging not associated to an app or social media platform.

| ☐ | Social Media Platform |
| --- | --- |
| ☐ | Online Discussion Forum |
| ☐ | Web or Smartphone application |
| ☐ | SMS messaging |
| ☐ | Other |

Number of Interventions

| ☐ | Online Peer Group is the only intervention |
| --- | --- |
| ☐ | Online Peer Group is part of multiple interventions |

If more than 1 intervention, describe below

|  |
| --- |

Did the online peer group include an active mediator?
Active mediators in the group include non-study participants such as researchers or health professionals, who participated in the group by initiating group discussions or answering/asking questions. N/A for non-interventional studies.

| ☐ | Mediator present |
| --- | --- |
| ☐ | No mediator present |
| ☐ | N/A |

**Duration of Intervention**
How long was the intervention provided?

|  |
| --- |

**Any other information** relating to the online peer support intervention
Describe the intervention, and how it was carried out if not already described above.

|  |
| --- |

## Participants

**Method of Recruitment**i.e. via Clinics, Online adverts, volunteer, invitation only

|  |
| --- |

**Population**
Based on which period the intervention was provided.
.i.e. provided during the prenatal period only or provided prenatally and postnatally.

| ☐ | Prenatal Only |
| --- | --- |
| ☐ | Prenatal and Postnatal |

Gestation and/or time post-birth
For prenatal studies what is the gestation period of participants? For studies that include postnatal population, what is the number of weeks/months post-birth?

|  |
| --- |

Primiparous or Multiparous
Primiparous - 1st Child. Multiparous - having more than 1 child. N/A if not stated.

| ☐ | Primiparous |
| --- | --- |
| ☐ | Multiparous |
| ☐ | Both |
| ☐ | N/A |

Total number of participants

|  |
| --- |

## Outcomes

How are outcomes measured?
i.e . What tools were used?

|  |
| --- |

How did the participants interact?
What details were given about how the participants interacted with each other? How much did participants engage in online peer groups? What kind of support did participants give and receive?

|  |
| --- |

What were the overall outcomes of the online peer group(s)?
For interventional studies only. Were outcomes for online peer groups positive? Was it useful or not useful for the intervention group? Were there differences in outcomes for control vs intervention groups?

|  |
| --- |

How did authors perceive outcomes/results of the study?
Extracted from the discussion section only. Did the author(s) discuss reasons why they have certain results? What was the author(s)'s interpretation of the results?

|  |
| --- |

## Types of social support demonstrated (Framework by Langford et al 1997)

**Emotional Support**
Emotional support involves the provision of caring, empathy, love and trust.
one (a) is cared for and loved, (b) is esteemed and valued, and (c) belongs to a network of mutual obligation.
‘subjective feeling of belonging, of being accepted, of being loved, of being needed..'

|  |
| --- |

**Instrumental support**
Provision of tangible goods and services, or tangible aid. Tangible aid is described as concrete assistance; for example, giving financial assistance or performing assigned work for others.

|  |
| --- |

**Informational Support**
information provided to another during a time of stress. informational support assists one to problem-solve.

|  |
| --- |

**Appraisal support**
Appraisal support involves the communication of information which is relevant to self-evaluation. Appraisal support as affirmational support. Affirmational support encompasses expressions that affirm the appropriateness of acts or statements made by another.

|  |
| --- |

**Other**
For other types of support that may not strictly fit into the Langford et al Social Support framework.

|  |
| --- |
