# Supplementary material for: The role of online peer support in pregnancy: A scoping review
Source: PLoS One. 2026 Jan 2;21(1):e0339876. doi: 10.1371/journal.pone.0339876 (PMC12758765; doi:10.1371/journal.pone.0339876)
Supplement: S4 File — (DOCX) [file pone.0339876.s004.docx]

**Supplementary file 3 Quality assessment of these studies in detail.**

**Table 3. 1 Overall Assessment using CASP Checklist for RCTs.**

|  | Duffecy et al., (2022) | Fiks et al.,(2017) | Leiferman et al., 2023 | Linden et al.,(2018) | Perera et al (2023) |
| --- | --- | --- | --- | --- | --- |
| Did the study address a clearly focused research question? | Y | Y | Y | Y | Y |
| Was the assignment of participants to interventions randomised? | Y | Y | Y | Y | Y |
| Were all participants who entered the study accounted for at its conclusion? | Y | Y | Y | Y | Y |
| Were the participants ‘blind’ to intervention they were given? | N | N | N | N | N |
| Were the investigators ‘blind’ to the intervention they were giving to participants? | Can’t tell | N | N | N | N |
| Were the people assessing/analysing outcome/s ‘blinded’? | Can’t tell | N | Can’t tell | Can’t tell | Can’t tell |
| Were the study groups similar at the start of the randomised controlled trial? | Y | Y | Can’t tell | Y | Y |
| Apart from the experimental intervention, did each study group receive the same level of care (that is, were they treated equally)? | Y | Y | Y | Y | Y |
| Were the effects of intervention reported comprehensively? | Y | Y | Y | Y | Y |
| Was the precision of the estimate of the intervention or treatment effect reported? | Y | Y | N | Y | Y |
| Do the benefits of the experimental intervention outweigh the harms and costs? | Y | Y | Y | N | Can’t tell |
| Can the results be applied to your local population/in your context? | Y | Y | Can’t tell | Y | Can’t tell |
| Would the experimental intervention provide greater value to the people in your care than any of the existing interventions? | Y | Can’t tell | Can’t tell | N | Can’t tell |
| OVERALL Quality | moderate | moderate | moderate | moderate | moderate |

**Table 3.2. Overall Assessment using JBI Critical Appraisal Checklist for QuasiExperimental Studies**

|  | Flax et al., (2022) | Jiang and Zhu., (2022a) | Salonen et al., (2014) | VanHaeken et al  2024 | Wu and Hung., (2019) |
| --- | --- | --- | --- | --- | --- |
| Is it clear in the study what is the ‘cause’ and what is the ‘effect’? | Y | Y | Y | Y | Y |
| Were the participants included in any comparisons similar? | Y | N | Y | N | Y |
| Were the participants included in any comparisons receiving similar treatment/care, other than the exposure or intervention of interest? | Y | N | Unclear | Y | Y |
| Was there a control group | Y | Y | Y | Y | Y |
| Were there multiple measurements of the outcome both pre and post the intervention/exposure? | Unclear | N | N | Y | Unclear |
| Was follow up complete and if not, were differences between groups in terms of their follow up adequately described and analyzed? | Y | N | Y | N | Y |
| Were the outcomes of participants included in any comparisons measured in the same way? | Y | Y | Y | Y | Y |
| Were outcomes measured in a reliable way? | Unclear | Unclear | Unclear | Y | N |
| Was appropriate statistical analysis used? | Y | Y | Y | Y | Y |
| OVERALL Quality | Moderate | Low | Moderate | Moderate | Moderate |

**Table 3. 4 Overall Assessment using CASP Checklist for Qualitative Research**

|  | Adler and Zarchin., (2002) | Dai et al., (2022) | Gleeson et al.,(2022) | Kouri et al., (2006) | Mattson & Ohlendorf., 2023 | McCarthy et al.,(2020) | Nguyen  (2023) | Shorey et al, 2022 |
| --- | --- | --- | --- | --- | --- | --- | --- | --- |
| Was there a clear statement of the aims of the research? | Y | Y | Y | Y | Y | Y | Y | Y |
| Is a qualitative methodology appropriate? | Y | Y | Y | Y | Y | Y | Y | Y |
| Was the research design appropriate to address the aims of the research? | Y | Y | Y | Y | Y | Y | Y | Y |
| Was the recruitment strategy appropriate to the aims of the research? | Y | Y | Y | Y | Y | Y | Y | Y |
| Was the data collected in a way that addressed the research issue? | Y | Y | Y | Y | Y | Y | Y | Y |
| Has the relationship between the researcher and participants been adequately considered? | N | N | Y | N | Y | N | N | N |
| Have ethical issues been taken into consideration? | Y | Y | Y | Y | Y | Y | Y | Y |
| Was the data analysis sufficiently rigorous? | Y | Y | Y | Y | Y | Y | Y | Y |
| Is there a clear statement of findings? | Y | Y | Y | Y | Y | Y | Y | Y |
| How valuable is the research? | Valuable | Valuable | Valuable | Valuable | Valuable | Valuable | Valuable | Valuable |
| OVERALL Quality | Moderate | Moderate | High | Moderate | High | Moderate | Moderate | Moderate |

**Table 3. 5 Overall Assessment using Mixed Methods Appraisal Tool**

|  | Amaro et al (2023)  Survey | Dean et al.,(2012) | Holtz et al., (2015)  (survey) | Jiang and Zhu., (2022b)  Survey | Lei et al.,(2022)   Online posts | Patel et al (2018) | Ronen et al., 2024 | Skelton et al.,(2020) | Simpson et al.,(2021) |
| --- | --- | --- | --- | --- | --- | --- | --- | --- | --- |
| S1.Are there clear research questions | N | Y | Y | Y | Y | Y | Y | Y | Y |
| S2. Do the collected data allow to address  the research questions? | Unclear | Y | Y | Y | Y | Y | Y | Y | Y |
| 1.1 Is the qualitative approach appropriate to answer the research question? | - | Y | - | - | - | Y | Y | Y | Y |
| 1.2 Are the qualitative data collection methods adequate to address the research question? | - | Y | - | - | - | Y | Y | Y | Y |
| Y1.3 Are the findings adequately derived from the data? | - | Can’t tell | - | - | - | Y | Y | Y | Y |
| 1.4 Is the interpretation of results sufficiently substantiated by data? | - | Y | - | - | - | Y | Y | Y | Y |
| 1.5 Is there coherence between qualitative data sources, collection, analysis and interpretation? | - | Y | - | - | - | Y | Y | Y | Y |
| 4.1. Is the sampling strategy relevant to address the research question? | Y | Y | Y | Y | Y | Y | Y | Y | Y |
| 4.2 Is the sample representative of the target population? | Y | Y | Y | Y | Y | Can’t tell | Can’t tell | Y | Y |
| 4.3. Are the measurements appropriate? | Y | Y | Y | Y | Y | Y | Y | Y | Y |
| 4.4. Is the risk of nonresponse bias low? | Y | Y | Y | Can’t tell | Can’t tell | N | Can’t tell | Y | Y |
| 4.5. Is the statistical analysis appropriate to answer the research question? | Y | N | Y | Y | Y | Y | Y | Y | Y |
| 5.1. Is there an adequate rationale for using a mixed methods design to address the research question? | - | Y | - | - | - | Y | Y | Y | Y |
| Y5.2. Are the different components of the study effectively integrated to answer the research question? | - | Y | - | - | - | Y | Y | Y | Y |
| 5.3. Are the outputs of the integration of qualitative and quantitative components adequately interpreted? | - | Y | - | - | - | Y | Y | Y | Y |
| 5.4. Are divergences and inconsistencies between quantitative and qualitative results adequately addressed? | - | (N/A) | - | - | - | Can’t tell | Y | (N/A) | (N/A) |
| -5.5. Do the different components of the study adhere to the quality criteria of each tradition of the methods involved? | - | Y | - | - | - | Y | y | Y | Y |
| OVERALL Quality | Moderate | Moderate | High | Moderate | Moderate | Moderate | Moderate | High | High |
