# Supplementary material for: The role of online peer support in pregnancy: A scoping review
Source: PLoS One. 2026 Jan 2;21(1):e0339876. doi: 10.1371/journal.pone.0339876 (PMC12758765; doi:10.1371/journal.pone.0339876)
Supplement: Attachment — Submitted filename: PONE-D-25-09522_feedback.docx [file pone.0339876.s005.docx]

Dear Editors and Authors,

Thank you very much for the opportunity to review this manuscript. I appreciate the editors considering me to review this manuscript. And congratulations to the authors for their work. This manuscript presents a critical study, but, there are some points that would benefit from further elaboration to enhance the paper's clarity.

**Feedback:**

1) Methods
   a. Please provide a brief explanation of the terms used in the search strategy and include the full search strategy as an appendix.
   b. It appears that the authors have included studies with various study designs. Please clarify this in the Methods section.
   c. Are there any limitations regarding the publication language or publication year? If so, please specify in the Methods section.
   d. How many individuals were involved in the methodological assessment process? Please provide this information in the Methods section.
   e. Was any specific software used in synthesizing the data? Additionally, how many individuals were involved in this process?
2) Regarding methodological assessment, most of the included studies were rated as having moderate quality. Could you provide guidance on how readers should interpret the results of this review in terms of the quality of the studies? Please report this in strengths and limitations.
3) Results:
   a. The description of the study selection process does not align with the PRISMA diagram. Please address this discrepancy
   b. Could you provide further details on the country context of the online peer support? Specifically, was it conducted in high-income countries? If so, please note this as a limitation, as the findings may be more applicable to high-income countries.
   c. Please ensure that studies are appropriately cited when reporting their characteristics.
   d. In eight studies, online peer support was combined with other interventions. How can we be certain that the observed effectiveness was specifically due to the online peer support and not other interventions?
4) What evidence supports the positive effects of peer support? Did the authors also include results from observational studies to make this determination? Additionally, what criteria were used to assess the effectiveness of peer support?
5) Could you clarify what other types of support are referred to in Table 3?
