# Supplementary material for: The role of online peer support in pregnancy: A scoping review
Source: PLoS One. 2026 Jan 2;21(1):e0339876. doi: 10.1371/journal.pone.0339876 (PMC12758765; doi:10.1371/journal.pone.0339876)
Supplement: Attachment — Submitted filename: Response to reviewers.docx [file pone.0339876.s007.docx]

| **Comments** | **Response** |
| --- | --- |
| **Editor comments** |  |
| PLOS requires an ORCID iD for the corresponding author in Editorial Manager on papers submitted after December 6th, 2016. Please ensure that you have an ORCID iD and that it is validated in Editorial Manager. | This has now been linked/added |
| Please ensure that you include a title page within your main document. You should list all authors and all affiliations as per our author instructions and clearly indicate the corresponding author. | This has now been added |
| Please ensure that you refer to Figure 1 in your text as, if accepted, production will need this reference to link the reader to the figure. | Reference to figure 1 added, p8 |
| Please include a caption for figure 1. | Caption added |
| Please include a copy of Table 2 which you refer to in your text on page 7 | Included |
| Please include captions for your Supporting Information files at the end of your manuscript, and update any in-text citations to match accordingly. | The supporting files now include captions and intext citations updated |
| Please include the period of the literature search in the abstract. Additionally, clarify the inclusion criteria with respect to study types (e.g., quantitative, qualitative, mixed methods) within the abstract’s methods section. | This has now been added |
| Please clarify or provide concrete examples of what is meant by "emotional support" to ensure consistent understanding among readers. | This has been added to abstract and methods sections (p7, l134) |
| While the review aims to explore the types, contexts, and effectiveness of online peer support, the findings primarily focus on factors influencing good conduct. Please provide a more comprehensive synthesis that also addresses the types of peer support and their effectiveness, or clarify the scope of the findings accordingly. | Our original aim included the term effectiveness but during the synthesis we found that effectiveness was rarely directly measured and could not be clearly linked to peer support alone. Many interventions included other components, making it hard to isolate the role of peer support.  Our synthesis has focused on mapping the types of support exchanged, the settings in which they occurred, and the structural factors that shaped group function. We have now clarified this more explicitly in the text by replacing references to effectiveness with perceive benefits to ensure alignment with the aims of a scoping review |
| Add clear subheadings for each methodological component, including: Study Selection, Data Extraction, Critical Appraisal Assessment (currently embedded and unclear, see around line 98). | Subheadings have been added |
| Clearly report if any software tools (e.g., NVivo, Excel) were used during data extraction or analysis. | Covidence data extraction tool was used, this is now included in methodology section |
| The manuscript reports the use of multiple appraisal tools (e.g., MMAT and others). Since MMAT covers RCTs, quasi-experimental, and qualitative studies, please justify why additional tools were needed despite MMAT’s broad applicability. Please also add critical appraisal results as supplementary files | While the MMAT was used for its flexibility in appraising diverse study designs, we also applied design-specific tools to increase appraisal depth and rigour. The CASP checklists (for qualitative and RCT studies) and the JBI checklist (for quasi-experimental studies) allowed us to assess features not covered by the MMAT, such as researcher reflexivity and outcome measurement timing. Full critical appraisal results are provided in the supplementary files. |
| Indicate the number of included studies by methodology (quantitative, qualitative, mixed methods) both in narratively in the results section and in the characteristics table. | This is noted in the narrative on page 9 second paragraph and in Table 2 |
| Report the geographic distribution of studies in results section (e.g., number from high-income vs. low- and middle-income countries). | This has been added, page 8 |
| Reference all tables and figures appropriately within the text (e.g., Figure 1 is uploaded but not cited). | This has been done, thank you |
| Use descriptive subheadings in the results section to guide readers especially before the findings synthesis section (e.g., “Overview of Included Studies,” “Types of Online Peer Support,” etc.). | This has been added |
| Ensure findings are consistently supported by appropriate references from the included studies. For example, in lines 187-192, citations should be added to show which included studies support the stated themes or observations. | These have been added, thank you |
| **Reviewer #1 comments** |  |
| Please provide a brief explanation of the terms used in the search strategy and include the full search strategy as an appendix. | There is a reference to the Prospero protocol which has the full search strategy.  Further explanation has been added to line 107-8. There is also a full search strategy now attached as an appendix |
| It appears that the authors have included studies with various study designs. Please clarify this in the Methods section. | This has been added to the inclusion/exclusion criteria |
| Are there any limitations regarding the publication language or publication year? If so, please specify in the Methods section. | Added into Methods p5 |
| How many individuals were involved in the methodological assessment process? Please provide this information in the Methods section | Added in to methods p5 |
| Was any specific software used in synthesizing the data? Additionally, how many individuals were involved in this process? | No specific software was used.  This was undertaken by two individuals. This has been clarified in the test |
| Regarding methodological assessment, most of the included studies were rated as having moderate quality. Could you provide guidance on how readers should interpret the results of this review in terms of the quality of the studies? Please report this in strengths and limitations. | We have now addressed this in the Strengths and Limitations section. We have suggested to interpret findings with caution, particularly regarding effectiveness. |
| **Results:** |  |
| The description of the study selection process does not align with the PRISMA diagram. Please address this discrepancy | Thank you. This has been corrected and now aligns |
| Could you provide further details on the country context of the online peer support? Specifically, was it conducted in high-income countries? If so, please note this as a limitation, as the findings may be more applicable to high-income countries. | The income of the country was only ever indirectly referenced in the articles. However, comment relating to the relevance of the findings to lower socioeconomic groups has been added to the discussion. |
| Please ensure that studies are appropriately cited when reporting their characteristics. | This has now been actioned.  Further explanation has been added to line 107-8. There is also a full search strategy now attached as an appendix |
| In eight studies, online peer support was combined with other interventions. How can we be certain that the observed effectiveness was specifically due to the online peer support and not other interventions? | Thank you for this insightful comment, as noted above it is true it is hard to distinguish what might have had the effect. That is in part why we did not explicitly assess effectiveness please see above in response to editor comment. We have added a section on this to the ‘Limitations’ |
| What evidence supports the positive effects of peer support? Did the authors also include results from observational studies to make this determination? Additionally, what criteria were used to assess the effectiveness of peer support? | As above, during the synthesis we found that effectiveness was rarely directly measured and could not be clearly linked to peer support alone. Many interventions included other components, making it hard to isolate the role of peer support. Our synthesis therefore focused on mapping the types of support exchanged, the settings in which they occurred, and the structural factors that shaped group function. |
| Could you clarify what other types of support are referred to in Table 3? | Types of support that may not strictly fit into the Langford et al Social Support framework. There were no studies that fell into this category and so it has been removed. |
| **Reviewer #2** |  |
| In the abstract, please state the method more precisely. Clearly indicate the inclusion criteria for the study and mention the databases used. | Thank you for this comment, we would like to add this but were unable to, due to the tight word count restriction for the abstract. |
| In the introduction, it is important to clearly articulate the necessity of conducting this research in the final paragraph. | We hope this is now clearly articulated |
| The discussion section could be strengthened. Please make the necessary revisions. | We are not clear what specifically was required as a revision in this comment, but believe that the discussion section adequately situates the findings in the wider literature/explores potential mechanisms of effect and provides a measured interpretation of the results. |
