# Supplementary material for: The role of online peer support in pregnancy: A scoping review
Source: PLoS One. 2026 Jan 2;21(1):e0339876. doi: 10.1371/journal.pone.0339876 (PMC12758765; doi:10.1371/journal.pone.0339876)
Supplement: Attachment — Submitted filename: Response to Reviewers November 2025.docx [file pone.0339876.s008.docx]

Response to Reviewers November 2025


Reviewer #2:

1. The necessity of the research is not clearly articulated in the abstract.
Response: We are sorry that the reviewer does not feel that the necessity for the research (review) is clear in the abstract, we have tried to make it as explicit as possible within the limited word count.
2. The methodology outlined in the abstract is incomplete. Please specify the databases used and include the inclusion and exclusion criteria.
Response: Thank you for this comment, we have added the databases, but been unable to include all criteria due to the word count restriction of the abstract.
3. The search strategy in the methodology should be detailed, including the keywords that were searched in the electronic databases.
Response: The detailed search strategy is in S2, which includes all the keywords
4. In Table 2, which presents the characteristics of the studies, please ensure that each study is referenced.
Response: Added, thank you
5. Consider adding a table to evaluate the quality of the studies.
Response: The quality appraisal stables are in S3
6. Strengthen the discussion section by providing an analysis of the studies.
Response: Thank you for this comment, we have provided analysis of the quality of the papers and provided a full synthesis, we do not feel that further individual analysis of each study is not appropriate in this type of review.
7. Lastly, please include the source of funding and any potential conflicts of interest at the end of your manuscript.
Response: The funding source is included on page 3 and a separate conflict of interest statement was submitted to the journal.

Reviewer #3:

1. Line 85: check spelling of 'excellence'.
Response: Corrected
2. Line 111: There is an extra 'e' and the fullstop is missing at the end.
Response: Corrected
3. Lines 172 & 275: Avoid starting sentences with a number.
Response: Corrected
Reviewer #4:
Overall assessment: This manuscript offers a timely and well-structured scoping review of online peer support in pregnancy, synthesizing 27 studies across diverse geographies and platforms. The mapping of findings to the Langford Conceptual Analysis of Social Support is a particular strength, helping to translate heterogeneous evidence into clear domains (emotional, informational, appraisal, instrumental). The review adds concrete design levers—group size, active moderation, and privacy settings—that have immediate relevance for program developers, clinicians, and digital health implementers. This work will make a valuable contribution to the maternal digital-health literature.
Thank you
General comments:
1. The authors alternate between “pregnant people’ and “pregnant women”. Clarify in the Introduction section if there is an intentional purpose for this. Otherwise, choose one term and apply it consistently throughout the manuscript.
Response: Thank you, this has been made consistent through the manuscript.
2. Line 85 – Fix the typo “Ecxellence” to “Excellence”.
Response: Corrected
3. Line 111 – The reference statement pointing to the supplemental document S2 ends abruptly. The authors should close out the sentence.
Response: Corrected
Line 129 – In addition to listing the data items considered in this scoping review, the authors should consider adding a Data Items Table and definitions (in supplementary materials) for reference.
Response: Thank you for this comment, we have included a new supplementary file which include the data extraction tool used (data items).
Line 188 – The reference against “Weight Management” is missing an opening bracket ( [ ). Ensure bracketed references are correct and consistent.
Response: Corrected
Line 257 – Consider using a dash (-) to proffer clarity for “9 13 people” as “9-13 people” if the intention is to portray a number range.
Response: Corrected
